# Surface antigens of the syphilis spirochete and their potential as virulence determinants.

**DOI:** 10.3201/eid0301.970102

**Published:** 1997

**Authors:** D R Blanco, J N Miller, M A Lovett

**Affiliations:** Dept. Microbiology and Immunology, UCLA School of Medicine 90095, USA. dblanco@microimmun.medsch.ucla.edu

## Abstract

A unique physical feature of Treponema pallidum, the venereally transmitted agent of human syphilis, is that its outer membrane contains 100-fold less membrane-spanning protein than the outer membranes of typical gram-negative bacteria, a property that has been related to the chronicity of syphilitic infection. These membrane-spanning T. pallidum rare outer membrane proteins, termed TROMPs, represent potential surface-exposed virulence determinants and targets of host immunity. Only recently has the outer membrane of T. pallidum been isolated and its constituent proteins identified. Five proteins of molecular mass 17-, 28-, 31-, 45-, and 65-kDa were outer membrane associated. The 17- and 45-kDa proteins, which are also present in greater amounts with the T. pallidum inner membrane protoplasmic cylinder complex, had been previously characterized lipoproteins and are, therefore, not membrane-spanning but rather membrane-anchored by their lipid moiety. In contrast, the 28-, 31-, and 65-kDa proteins are exclusively associated with the outer membrane. Both the purified native and an Escherichia coli recombinant outer membrane form of the 31-kDa protein, designated Tromp1, exhibit porin activity, thereby confirming the membrane-spanning outer membrane topology of Tromp1. The 28-kDa protein, designated Tromp2, has sequence characteristics in common with membrane-spanning outer membrane proteins and has also been recombinantly expressed in E. coli, where it targets exclusively to the E. coli outer membrane. The 65-kDa protein, designated Tromp3, is present in the least amount relative to Tromps1 and 2. Tromps 1, 2, and 3 were antigenic when tested with serum from infection and immune syphilitic rabbits and humans. These newly identified TROMPs provide a molecular foundation for the future study of syphilis pathogenesis and immunity.


### Surface Antigens of the Syphilis Spirochete and Their Potential as Virulence Determinants

**David R. Blanco, James N. Miller, and Michael A. Lovett**
UCLA School of Medicine, Los Angeles, California

A unique physical feature of *Treponema pallidum*, the venereally transmitted agent of human syphilis, is that its outer membrane contains 100-fold less membrane-spanning protein than the outer membranes of typical gram-negative bacteria, a property that has been related to the chronicity of syphilitic infection. These membrane-spanning *T. pallidum* rare outer membrane proteins, termed TROMPs, represent potential surface-exposed virulence determinants and targets of host immunity. Only recently has the outer membrane of *T. pallidum* been isolated and its constituent proteins identified. Five proteins of molecular mass 17-, 28-, 31-, 45-, and 65-kDa were outer membrane associated. The 17- and 45-kDa proteins, which are also present in greater amounts with the *T. pallidum* inner membrane protoplasmic cylinder complex, had been previously characterized lipoproteins and are, therefore, not membrane-spanning but rather membrane-anchored by their lipid moiety. In contrast, the 28-, 31-, and 65-kDa proteins are exclusively associated with the outer membrane. Both the purified native and an *Escherichia coli* recombinant outer membrane form of the 31-kDa protein, designated Tromp1, exhibit porin activity, thereby confirming the membrane-spanning outer membrane topology of Tromp1. The 28-kDa protein, designated Tromp2, has sequence characteristics in common with membrane-spanning outer membrane proteins and has also been recombinantly expressed in *E. coli,* where it targets exclusively to the *E. coli* outer membrane. The 65-kDa protein, designated Tromp3, is present in the least amount relative to Tromps1 and 2. Tromps 1, 2, and 3 were antigenic when tested with serum from infection and immune syphilitic rabbits and humans. These newly identified TROMPs provide a molecular foundation for the future study of syphilis pathogenesis and immunity.

Despite the fact that *Treponema pallidum* subsp. *pallidum* has remained sensitive to penicillin for more than four decades, syphilis remains an important cause of sexually transmitted disease, accounting for more than 14,000 new cases in 1995 in the United States alone (1). Although syphilis is not an emerging infectious disease, its reemergence, from 1986 to 1990, culminated in more than 100,000 reported cases in 1990 and an 80% increase in the reported rate per 100,000 population. The reemergence of syphilis has also been associated in certain areas with the use of crack cocaine, and syphilis continues to be an important risk factor for acquiring and transmitting human immunodeficiency virus.

Syphilis is characterized by months of clinical disease followed by years of latency with the potential for relapse to debilitating or lethal late disease if left untreated. The chronicity of infection may relate to a striking property of the *T. pallidum* outer membrane, namely that it contains 100-fold less membrane-spanning protein than the outer membranes of typical gram-negative bacteria (2,3). This article reviews recent progress in identifying and characterizing the rare outer membrane proteins (TROMPs) of *T. pallidum*.

The interaction of host and pathogen leading to the establishment of infection generally proceeds through the interactions of their respective surface molecules. An understanding of the molecular basis for syphilis pathogenesis and the identification of corresponding *T. pallidum* surface molecules have long been hampered by the inherent difficulties associated with the study of this organism. In contrast to that of many other pathogenic spirochetes from the genera *Treponema*, *Borrelia*, and *Leptospira*, the in vitro multiplication of *T. pallidum* is unsuccessful in artificial media and is limited and impractical in tissue culture (4). Further, only limited numbers of *T. pallidum* are

Address for correspondence: David R. Blanco, Dept. Microbiology and Immunology, CHS 43-239, UCLA School of Medicine, 10833 LeConte Ave., Los Angeles, CA 90095; fax:310-206-3865; e-mail: dblanco@microimmun.medsch.ucla.edu.

attainable from testicular cultivation in rabbits. Organisms acquired from rabbit infection are also bound with host material including albumin (5), fibronectin (6), and glycosaminoglycans (7); therefore, the separation and purification of *T. pallidum* molecules are complicated. Host material associated with *T. pallidum* has also been the basis for past theories of molecular mimicry during syphilitic infection (6), of *T. pallidum* adherence to host cells and tissues (8), and of the long recognized antigenic inertness of the surface of this organism.

Past studies showed that the surface of *T. pallidum* behaves as if it lacks proteins and antigens. These findings included the inability to detect antibody bound to the surface of structurally intact treponemas by immunofluorescence (9) or by immunoelectron microscopy (10,11), the limited radiolabeling of surface proteins (12,13), and the observation that only with extended in vitro incubation could proteins of intrinsically radiolabeled *T. pallidum* bind specific antibody (14). One hypothesis generated by these observations was that the surface of *T. pallidum* was antigenically inert because of a limited number of available surface-exposed antigens.

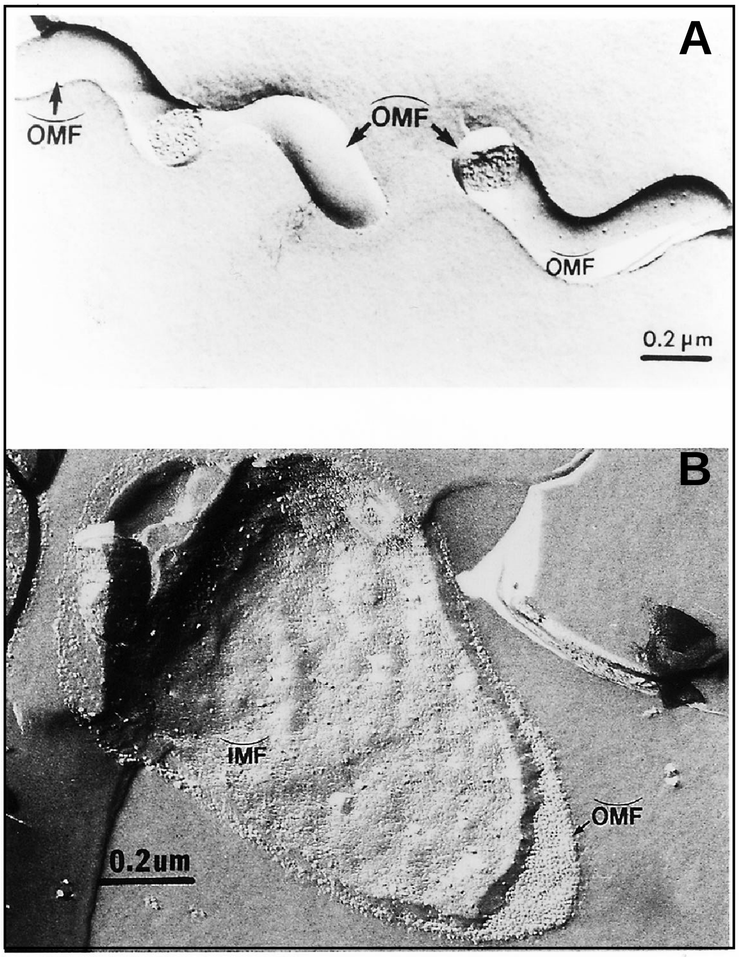

Figure 1. Freeze-fracture electron microscopy of *Treponema pallidum* subsp. *pallidum* (A), and *Escherichia coli* (B). Concave ( ) and convex ( ) outer membrane (OMF) and inner membrane (IMF) fracture faces.

The inability to apply conventional gram-negative cell fractionation procedures to *T. pallidum* to isolate its outer membrane and identify its constituent proteins further hampered the identification of surface-exposed proteins. As a result, alternative methods, which included detergent solubilization, were used to achieve this goal. The detergent Triton X-114 (TX-114) was used with particular interest because of its inherent property of a low temperature cloud point that results in phase separation yielding both aqueous and detergent phases. It was found that 0.1% to 2% TX-114 would selectively solubilize the *T. pallidum* outer membrane and result in the recovery of several prominent detergent-phase proteins (15,16), which were speculated, at the time, to be part of the outer membrane and potentially surface exposed. These proteins were subsequently found to be lipoproteins (17) and were strongly antigenic when tested with serum from syphilitic rabbits and humans (17). While surface exposed lipoproteins have been demonstrated for other pathogenic spirochetes, including *Borrelia* (18) and *Leptospira* (19), a surface exposed location for a strongly antigenic *T. pallidum* lipoprotein was inconsistent with the surface antigenic inertness of this organism.

A major advance in the understanding of the *T. pallidum* outer membrane and cell surface followed the results of freeze-fracture electron microscopy in 1989. The observation that the fracture faces of the *T. pallidum* outer membrane contained only 170 particles/$\mu m^2$ (2,3), which is approximately 100-fold less than what is contained in typical gram-negative bacterial outer membranes (Figure 1), together with the finding of freeze-etch analysis (3), has provided the most plausible explanation for the antigenic inertness of the *T. pallidum* surface. The rare particles revealing *T. pallidum* rare outer membrane proteins were designated TROMPs (2,20), and because of their membrane-spanning topology, they were distinct from lipoproteins, which do not span membranes to form particles in freeze-fracture analyses (21). Moreover, a membrane-spanning protein by definition should possess several surface exposed regions, suggesting that TROMPs had surface exposed antigenic sites. This theory was confirmed by freeze-fracture analysis when it was observed that incubation of *T. pallidum* in serum from syphilitic rabbits immune to reinfection resulted in the aggregation of TROMPs (20) (Figure 2). It was found that 8 to 16 hours of incubation was required for this aggregation to occur, suggesting that antibody-mediated aggregation of TROMPs is the rate limiting step for complement activation and killing of *T. pallidum* (20).

The findings of a potentially protective immunogen in low molar amounts on the outer membrane surface of *T. pallidum* have provided a further attractive explanation for the prolonged period required for the induction of acquired protective immunity during syphilitic infection. This is also in accord with the repeated immunizations required for protective immunity with *T. pallidum* attenuated by gamma irradiation (22), the only method of successful vaccination ever reported. Thus, the identification and study of TROMPs has become essential in understanding syphilis pathogenesis and immunity in molecular terms.

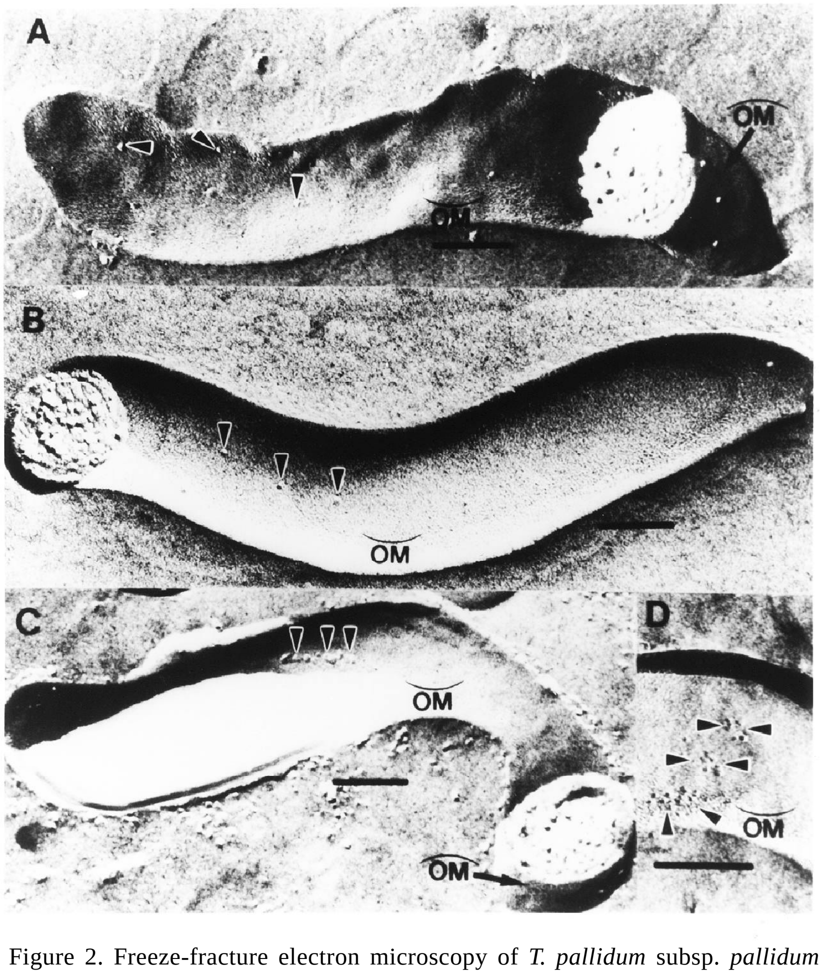

Figure 2. Freeze-fracture electron microscopy of *T. pallidum* subsp. *pallidum* demonstrating TROMP aggregation. Concave ( ) and convex ( ) outer membrane fracture faces (OM). *T. pallidum* incubated in heat-inactivated normal rabbit serum for 16 hours (A), in immune rabbit serum for 2 hours (B), and in immune rabbit serum for 16 hours (C) and (D). Arrows show individual (A&B) and aggregated (C&D) TROMPs. Bar in each micrograph represents 0.1µm. Photograph reprinted with the permission of the Journal of Immunology, copyright 1990, The American Association of Immunologists.

### Isolation of the *T. Pallidum* Outer Membrane

In 1994, we developed a procedure to isolate the outer membrane of *T. pallidum* without detergents (23). This method used three key features: 1) a Ficoll step gradient to purify *T. pallidum* from host contaminating material; 2) octadecyl rhodamine B chloride salt, a lipid-conjugated chromophore that intercalates into membranes and provides a visual marker to monitor outer membrane release and recovery; and 3) a hypotonic 0.05 M sodium citrate buffer, pH 3.0, which was found to selectively release the *T. pallidum* outer membrane. Since it has been suggested that endoflagellar filaments physically interact with the outer membrane in the process of motility, the probable mechanism for outer membrane release by this procedure is the depolymerization of endoflagella at low pH.

Purification of *T. pallidum* outer membrane vesicles in sucrose density gradients showed that the membrane banded at the very low density of 1.03 g/ml (7% sucrose). This, however, was expected for properties of a membrane that lacks lipopolysaccharide (24) and contains a small amount of protein. Freeze-fracture electron microscopy demonstrated that the purified membrane vesicles contained extremely rare intramembranous particles consistent with the low particle density observed for the native outer membrane of *T. pallidum*. The selective isolation of the *T. pallidum* outer membrane was shown by the inability to detect *T. pallidum* penicillin binding proteins, a marker of the cytoplasmic membrane. Further support for the selectivity of the outer membrane isolation was the complete absence of a previously characterized 19-kDa periplasmic protein termed 4D (15) and the abundant 47-kDa lipoprotein (17) and the presence of only trace amounts of endoflagellar protein. The absence of the 47-kDa lipoprotein was particularly important since it is widely accepted that this very abundant protein is exclusively anchored to the inner membrane (25). The finding that the 47-kDa lipoprotein was not detected in the purified outer membrane material indicates that inner membrane anchored lipoproteins were not released by this procedure.

Two dimensional immunoblot analysis was used to identify the protein constituents present in purified *T. pallidum* outer membrane. Two strongly antigenic species were found by syphilitic immune rabbit serum, one very basic protein (pI > 7.0) at 17-kDa and the other having a pI of approximately 4.5 at 45-kDa. The observation that the 17-kDa protein was very basic, showed higher oligomeric forms, and was selectively partitioned into the hydrophobic phase after Triton X-114 detergent extraction was consistent with properties reported for the 17-kDa lipoprotein of *T. pallidum* (26). With specific monoclonal antibodies, the 45-kDa protein was identified as the previously characterized lipoprotein termed TmpA (27). It was also found that most of these two lipoproteins, well over 90%, are associated with the inner membrane protoplasmic cylinder complex. It would appear, however, that the association of the 17- and 45-kDa lipoproteins with the outer membrane is specific, given the absence of the normally abundant 47-kDa lipoprotein. The function of these two outer membrane lipoproteins remains to be determined.

In addition to the strongly antigenic 17- and 45-kDa lipoproteins, gold stained two-dimensional blots of outer membrane showed four additional *T. pallidum* proteins, including one each at 28- and 65-kDa and two at 31-kDa, with close but distinct pI migration patterns. All of these proteins, including the 17- and 45-kDa lipoproteins, were present in approximately equal amounts in the outer membrane. However, in contrast to the 17- and 45-kDa lipoproteins, these four additional proteins required very low dilutions (1:25) of immune syphilitic rabbit serum for detection by immunoblot analysis. Moreover, when compared with stained two-dimensional blots of total *T. pallidum* proteins, these four additional outer membrane proteins were either not detectable, as was the case for the 65-kDa protein and the more basic of the two 31-kDa proteins, or represented extremely minor species, as was the case for the 28-kDa protein and the more acidic 31-kDa protein. In view of the paucity of TROMPs determined from freeze-fracture analysis, these four proteins, which were clearly enriched in our outer membrane preparation, became the leading candidates for membrane-spanning outer membrane proteins of *T. pallidum*.

In 1995, a second *T. pallidum* outer membrane isolation method that used plasmolysis of organisms in 20% sucrose was reported by Radolf et al. (28). Outer membrane isolated by this method was also shown by freeze-fracture analysis to have an extremely low density of membrane-spanning protein. These studies showed that the lipid composition of the *T. pallidum* outer membrane was similar to that of the protoplasmic cylinder inner membrane complex, with the exception of cardiolipin, which was prominently detected only in protoplasmic cylinders. Of further interest was the finding that outer membrane phospholipids and glycolipids did not react with antibody from infection-derived immune serum, suggesting that only outer membrane proteins are target antigens. However, in contrast to the limited number of protein species that we observed, silver stained gels of their outer membrane material showed more than 20 proteins, including the 47-kDa lipoprotein. One interpretation of these results is that many of the proteins identified in their outer membrane preparation are possibly contaminants of the periplasm or inner membrane. Thus, we believe that the small number of protein species identified by our isolation method represent a more accurate account of the outer membrane protein composition of *T. pallidum*.

While lipoproteins are integral membrane proteins, their membrane association extends only to integration of the lipid moiety in the bilayer. In contrast to membrane-spanning proteins, the *T. pallidum* lipoproteins do not span membrane lipid bilayers and, therefore, do not form particles upon freeze-fracture electron microscopy (21). By comparison, one type of membrane-spanning protein common to virtually all gram-negative outer membranes are porins, which form water-filled channels through membranes allowing for transport of small molecular weight solutes (29). Porin proteins, like almost all outer membrane proteins, span membranes in a beta-pleated sheet topology rather than in alpha helical hydrophobic regions, which are common to inner membrane-spanning proteins (30). Porin activity can be detected by several methods (29), including radioisotope efflux or substrate uptake in proteoliposomes, liposome swelling, and the black lipid bilayer assay, which measures the conductivity of ions through porin channels. In the black lipid bilayer assay, a set voltage is applied across an artificial lipid bilayer which, if a porin protein has been inserted, results in an increase in electrical conductance. The degree of conductance increase for many insertional events is used to calculate the average pore channel size. When the black lipid bilayer assay was used with Triton X-100 detergent solubilized proteins from our purified *T. pallidum* outer membrane, porin activity was demonstrated (23). Two distinct average conductance increases were observed at 0.4 and 0.76 nanosiemens (nS), corresponding to pore channel sizes of 0.35 and 0.68 nm, respectively, which are similar in size to porin channels found for other gram-negative bacteria (29). At the time, the two distinct conductance measurements suggested two different porin species, or alternatively, that the larger activity could be the result of dimeric aggregates of the smaller activity or the smaller activity could be a substate of the larger channel, possibly caused by the application of a voltage (31). Regardless of the exact number of *T. pallidum* porin species, these findings confirmed that at least some of the particles observed by freeze-fracture electron microscopy of the *T. pallidum* outer membrane were membrane-spanning porin proteins. This finding was of particular importance since several gram-negative bacterial porins play a role in pathogenesis by acting as adhesins (32,33) and as surface targets of bactericidal antibody (34-37). Thus, the search for an outer membrane *T. pallidum* porin protein was initiated to identify a first TROMP species.

### *T. Pallidum* Rare Outer Membrane Protein 1 (Tromp1): an Outer Membrane Porin

Since most porin proteins have molecular masses of 28 to 48-kDa (29), our focus was directed toward isolating the 28- and 31-kDa proteins identified in purified *T. pallidum* outer membranes. Internal amino acid sequences from the native 31-kDa protein were used to clone the encoding structural gene, designated tromp1 (38). Antiserum generated to a recombinant Tromp1 fusion protein has shown that both 31-kDa proteins identified by two-dimensional blot analysis of outer membrane are Tromp1. Analysis of the deduced amino acid sequence showed an N-terminal hydrophobic region consistent with a signal peptide. Two potential leader peptidase I cleavage sites are noted, including threonine-histidine-alanine at residues 30 through 32 and alanine-alanine-alanine at residues 38 through 40. Identification of the cleavage site by N-terminal amino acid sequence of the native protein has not been possible because of the limited amount of native protein recoverable. However, other data discussed later in this review suggest that alanine-alanine-alanine is the correct cleavage site. The deduced Tromp1 amino acid sequence also supports the concept that Tromp1 topology is in accord with the structural paradigms of other gram-negative outer membrane proteins. Beta-moment analysis, which shows amphipathic sequence regions, has predicted that Tromp1 has 14 membrane-spanning amphipathic beta-sheet segments typical of gram-negative outer membrane proteins (29,30).

The lethality for *Escherichia coli* transformants harboring the intact tromp1 structural gene, which was found by immunoblot analysis to be expressing Tromp1, is similar to that observed for many recombinant gram-negative porin proteins expressed in *E. coli* (39). The tromp1 gene has recently been found to be part of a putative transport operon that includes an adenosine triphosphate binding protein and a hydrophobic membrane protein (Hardham et al., Gordon Research Conference, 1996). To determine if Tromp1 was a porin, native Tromp1 was purified from Triton X-100 solubilized *T. pallidum* by nondenaturing isoelectric focusing. The demonstration of porin activity, by the black lipid bilayer assay, has confirmed that Tromp1 is a membrane-spanning outer membrane protein of *T. pallidum*, the first such protein to be identified for this organism. Native Tromp1 showed two distinct conductance distributions about means of 0.15 and 0.7 nS. The larger of these channels is similar in mean conductance to the 0.76 nS activity observed by using purified outer membrane. The smaller 0.15 nS conductance channel had not been detected previously, and while two channels of different sizes have been reported for other porins (40), this may simply be the result of partial damage during isolation.

Because most gram-negative bacterial porins exist in the outer membrane in either a sodium dodecyl sulfate (SDS) unstable or stable trimer conformation (29), it was theorized that Tromp1 would also be organized in an oligomeric form in the outer membrane of *T. pallidum*. Antiserum against a Tromp1 fusion protein has in fact identified two higher molecular mass forms of native Tromp1 on immunoblots of *T. pallidum* with sizes of approximately 55- and 80-kDa. A 98-kDa size is further found to be the only detectable form of native Tromp1 when *T. pallidum* is solubilized at room temperature in a low concentration (0.02%) of SDS. These findings would appear to be consistent with the idea that native Tromp1 exists in the *T. pallidum* outer membrane in an SDS-unstable trimer conformation, a property that would be common to several other gram-negative outer membrane porins (29).

### Expression of a Porin Active Recombinant Tromp1 (rTromp1)

Porin proteins of gram-negative pathogens not only function as portals for nutrient acquisition across the outer membrane but also play a role in pathogenesis by acting as adhesins (32,33) and targets for bactericidal antibody (34-37). The membrane-spanning conformation of membrane porins is critical for their biologic function. Many surface-exposed epitopes on porins shown to be targets for bactericidal antibodies are conformational (34,36). Thus, correct outer membrane protein conformation should be a key consideration in the study of porins as they relate to bacterial virulence and host immunity. The previous demonstration of immune serum antibody mediated aggregation of TROMPs, as viewed by freeze-fracture electron microscopy (20), suggested that Tromp1 could be a key surface exposed target for antibody that mediates killing (20,41) or opsonization (42,43) of *T. pallidum*. Because of these possibilities, studies have been initiated to express and isolate a porin form of recombinant Tromp1 (rTromp1) with native conformation and in amounts necessary to conduct functional studies.

The controlled nonlethal expression in *E. coli* of exported rTromp1 was accomplished by inserting the tromp1 gene, including the region encoding its native signal peptide, into a relatively low copy number expression plasmid that has an inducible T7 promoter (44). Uninduced basal levels of T7 RNA polymerase resulted in the stable expression, export, and outer membrane localization of rTromp1. Expression detected in whole cell lysates showed several forms of rTromp1, ranging in molecular mass from 31- to 35-kDa. These different sizes of rTromp1 may represent different conformations of the protein as observed for other recombinant outer membrane proteins expressed in *E. coli*. Only the 35-kDa form of rTromp1 is detected in outer membranes isolated from *E. coli*. One explanation for the higher molecular mass outer membrane form is its possible association with lipopolysaccharides, known to form a complex with gram-negative outer membrane porin proteins (45). Such an interaction would certainly be noteworthy because the *T. pallidum* outer membrane does not contain lipopolysaccharides (24).

The demonstration of porin activity by using rTromp1 isolated from *E. coli* outer membranes has further confirmed the membrane-spanning conformation of Tromp1. Single channel conductance measurements of rTromp1 in the black lipid bilayer assay showed two distinct distributions of activity about 0.4 and 0.8 nS. The 0.8 nS conductance is clearly similar to the 0.7 nS conductance determined for native Tromp1 (38) and the 0.76 nS conductance determined for purified outer membrane, indicating that the pore-forming conformation of a portion of rTromp1 is similar if not identical to that of the native protein. Although it is not known why rTromp1 also showed a 0.4 nS conductance measurement, one possibility is an altered conformation of the protein after the isolation process.

A further question regarding the outer membrane localization of porin active rTromp1 has been whether surface exposed antigenic sites are present. Whole mount immunoelectron microscopy has demonstrated the surface binding of immune rabbit serum antibody on *E. coli* expressing rTromp1 (44). Antibody raised against a soluble fusion protein form of rTromp1, which was found by immunoblot analysis to be 100-fold more sensitive in detecting Tromp1 than the immune rabbit serum antibody, did not show surface binding of antibody on *E. coli* expressing rTromp1. This soluble fusion protein form of rTromp1 does not have porin activity when tested in the black lipid bilayer assay. Thus, a reasonable explanation for the immunoelectron microscopy observations is that the bound immune rabbit serum antibodies recognize conformational surface epitopes on rTromp1, a possibility which may also extend to native Tromp1 on the surface of *T. pallidum*.

The importance of porin protein conformation to biologic function has been further demonstrated in studies using rTromp1. The primary amino acid sequence of Tromp1 suggests two possible signal peptide processing sites within the first 40 residues at threonine-histidine-alanine and at alanine-alanine-alanine. Signal peptide fusion constructs at these two possible cleavage sites using the OmpT signal peptide of *E. coli* result in both forms of rTromp1 being exported and targeted to the *E. coli* outer membrane. Only the OmpT signal fused at the alanine-alanine-alanine position resulted in an exported product that has a molecular mass closest to that of native Tromp1. Moreover, while the outer membrane form of rTromp1 processed at threonine-histidine-alanine showed porin activity, the average channel conductance was 3.2 nS, which is considerably larger than the 0.7-0.8 nS channels observed for native and recombinant Tromp1 exported with its native signal peptide. The greater lethality and limited recovery of the OmpT-Tromp1 fusion construct processed at alanine-alanine-alanine has precluded at this time the ability to test this outer membrane form for porin activity. These findings suggest that alanine-alanine-alanine is the likely processing site in the Tromp1 sequence for leader peptidase I. More importantly, these findings have indicated that subtle changes in the length of the primary amino acid sequence of Tromp1 can have significant effects upon porin activity and are likely the result of an altered conformation of the protein. For this reason and as described previously, proper Tromp1 conformation is an important consideration for future studies addressing its role in syphilis pathogenesis.

### *T. Pallidum* Rare Outer Membrane Protein 2 (Tromp2)

In the search for additional TROMP species, we have recently focused on the 28-kDa protein identified in purified *T. pallidum* outer membrane preparations (23). As in the protein for Tromp1, internal amino acid sequences from the native 28-kDa protein were used to clone the encoding structural gene, now designated tromp2 (46). Analysis of the deduced amino acid sequence showed a 24-residue N-terminal hydrophobic region consistent with a signal peptide and terminating in a typical leader peptidase I cleavage site of leucine-alanine-alanine. As for Tromp1, the deduced amino acid sequence for Tromp2 is also in accord with the structural paradigms of other gram-negative outer membrane proteins. Beta-moment analysis has predicted that Tromp2 has 9 membrane-spanning amphipathic beta-sheet segments.

Recombinant expression and export of Tromp2 (rTromp2) in *E. coli* has recently been accomplished by using the entire tromp2 structural gene in a relatively low copy number plasmid that has an inducible T7 promoter (46). In contrast to Tromp1, Tromp2 expression was not found to be lethal to *E. coli*, even under maximum inducing conditions. Immunoblot analysis using antiserum generated to rTromp2 has shown that virtually all of the recombinant protein produced is targeted to the *E. coli* outer membrane. When tested in the black lipid bilayer assay, rTromp2 isolated from *E. coli* outer membranes showed only occasional channel formation. The total number of porin insertional events was extremely low when compared with the amount of rTromp2 tested. This is in contrast to both native and recombinant Tromp1 where numerous insertional porin events were readily observed. Thus, whether Tromp2 truly has porin function is not clear at this time.

As for rTromp1, a question regarding the outer membrane localization of rTromp2 was the existence of surface exposed antigenic sites. The use of whole mount immunoelectron microscopy demonstrated the surface binding of immune rabbit serum antibody on *E. coli* expressing rTromp2 (46). By comparison, antibody raised against a denatured form of rTromp2, which by immunoblot analysis was found to be more sensitive in detecting rTromp2 than immune rabbit serum, did not show surface binding on *E. coli* expressing rTromp2. This finding is again similar to that described above for rTromp1 and again suggests that the bound immune rabbit serum antibodies recognize conformational-surface epitopes on rTromp2, a possibility which may extend to native Tromp2 on *T. pallidum*.

### *T. Pallidum* Rare Outer Membrane Protein 3 (Tromp3)

Of the three outer membrane protein species identified (28-, 31-, and 65-kDa), the 65-kDa protein, tentatively termed Tromp3, is present in the least amount on the basis of 2-dimensional SDS-PAGE gold-stained immunoblots of purified outer membrane (23). In addition, the presence of the 65-kDa protein from one outer membrane preparation to the next has been inconsistent, suggesting that this protein may be differentially expressed. The extremely small amount of Tromp3 has at this time precluded amino acid sequencing and, therefore, the cloning of the gene that encodes this protein.

### The Potential Role of TROMPs in Acquired Immunity Against Syphilis

Freeze-fracture electron microscopy has demonstrated the ability of serum from immune syphilitic rabbits to aggregate TROMPs (20). These studies have been extended by using serum from infected rabbits with varying degrees of challenge immunity and have shown that TROMP aggregation correlates directly with the development of challenge immunity (Lewinski et al., unpub. obs., 1994). These findings are in accord with past studies that have demonstrated a significant to complete level of protection of animals after passive immunization with serum from immune donors (47). In a recent study, we have used the small amount of attainable *T. pallidum* outer membrane to immunize mice. We have found that serum from the immunized mice possesses complement-dependent treponemicidal activity (100% killing of *T. pallidum*) that is 30 times greater than that of serum from immune syphilitic rabbits (Blanco et al., unpub. obs.). Immunoblot analysis using this serum has shown that only the outer membrane associated proteins are detected. We have also found that absorption of this serum to remove antibodies to the *T. pallidum* lipoproteins does not diminish the titer of the 100% killing activity. Such levels of serum treponemicidal activity have heretofore never been generated by immunization of mice or rabbits with either native or recombinant *T. pallidum* antigens. These observations are consistent with the idea that TROMPs represent the key surface exposed targets for treponemicidal antibody and perhaps a protective host immune response.

Our recent ability to express in sufficient amounts recombinant Tromp1 and Tromp2 provides an opportunity to address directly the ability of these *T. pallidum* outer membrane proteins to elicit protective immunity in experimental animals. However, the correct outer membrane conformation of the recombinant TROMPs may prove a key factor in whether protective immunity results, as is the case for several bacterial porins. We hope that TROMP immunization will generate a humoral and/or cellular response capable of efficiently killing *T. pallidum* and provide a significant level of acquired resistance.

### Acknowledgments

We thank Dr. Eldon M. Walker for the electron microscopy presented in this review and Dr. Cheryl I. Champion for her extensive contributions to the studies involving Tromp1 and Tromp2.

This paper is dedicated to Dr. Mary C. Pangborn, the discoverer of cardiolipin at the Division of Laboratories and Research, New York State Department of Health, for her 90th birthday, and to Dr. Thomas B. Turner, the pioneer of treponemal research conducted at the Johns Hopkins School of Medicine, for his 100th birthday.

Dr. Blanco is a member of the Department of Microbiology and Immunology, UCLA School of Medicine, Los Angeles. He has been involved over the past 16 years in syphilis research focusing on structure-function relationships and the immunobiology of surface and subsurface proteins.

### References

1. Centers for Disease Control and Prevention. Cases of selected notifiable diseases. MMWR Morb Mortal Wkly Rep 1995;44:917.
2. Walker EM, Zamphigi GA, Blanco DR, Miller JN, Lovett MA. Demonstration of rare protein in the outer membrane of *Treponema pallidum* subp. *pallidum* by freeze-fracture analysis. J Bacteriol 1989;171:5005-11.
3. Radolf JD, Norgard MV, Shulz WW. Outer membrane ultrastructure explains the limited antigenicity of virulent *Treponema pallidum*. Proc Natl Acad Sci USA 1989;86:2051-5.
4. Norris SJ, Edmondson DG. Factors affecting the multiplication and subculture of *Treponema pallidum* subsp. *pallidum* in a tissue culture system. Infect Immun 1987;53:534-9.
5. Alderete JF, Baseman JB. Surface-associated host proteins on virulent *Treponema pallidum*. Infect Immun 1979;26:1048-56.
6. Baughn RE. Role of fibronectin in the pathgenesis of syphilis. Reviews of Infectious Diseases 1987;9:372-85.
7. Fitzgerald TJ, Johnson RC. Surface mucopolysaccharides of *Treponema pallidum*. Infect Immun 1979;24:244-51.
8. Baseman JB, Alderete JF, Freeman-Shade L, Thomas DD, Peterson KM. Adhesin-receptor recognition between the syphilis spirochete and fibronectin. In: Leive L, editor. Microbiology 1986. Washington, (DC): American Society for Microbiology, 1986.
9. Deacon WE, Falcone VH, Harris A. A fluorescent test for treponemal antibodies. Proc Soc Exp Biol Med 1957;96:477-80.
10. Radolf JD, Fehniger TE, Siverblatt F, Miller JN, Lovett MA. The surface of virulent *Treponema pallidum*: resistance to antibody binding in the absence of complement and surface association of recombinant antigen 4D. Infect Immun 1986;52:579-85.

11. Hovind-Hougen K, Birch-Andersen A, Nielsen HA. Electron microscopy of treponemes subjected to the *Treponema pallidum* immobilization (TPI) test. APMS 1979;87:263-8.
12. Penn CW, Cockayne A, Bailey MJ. The outer membrane of *Treponema pallidum*: biological significance and biochemical properties. Journal of General Microbiology 1985;131:2349-57.
13. Norris SJ, Sell S. Antigenic complexity of *Treponema pallidum*: antigenicity and surface localization of major plypeptides. J Immunol 1984;133:2686-92.
14. Stamm SV, Hokinka RL, Wyrick PB, Bassford PJ. Changes in the cell surface properties of *Treponema pallidum* that occur during in vitro incubation of freshly extracted organisms. Infect Immunol 1987; 55:2255-61.
15. Cunningham TM, Walker EM, Miller JN, Lovett MA. Selective release of the *Treponema pallidum* outer membrane and associated polypeptides with triton X-114. J Bacteriol 1988;170:5789-96.
16. Radolf JD, Chamberlain NR, Clausell A, Norgard MV. Identification and localization of integral membrane proteins of virulent *Treponema pallidum* by phase partitioning with the nonionic detergent triton X-114. Infect Immun 1988;56:490-8.
17. Chamberlain NR, Brandt ME, Erwin AL, Radolf JD, Norgard MV. Major integral membrane protein immunogens of *Treponema pallidum* are proteolipids. Infect Immun 1989;57:2872-7.
18. Bergstrom S, Bundoc VG, Barbour AG. Molecular analysis of linear plasmid-encoded major surface proteins, OspA and OspB, of the Lyme disease spirochaete *Borrelia burgdorferi.* Mol Microbiol 1989;3:479-86.
19. Shang ES, Summers TA, Haake DA. Molecular cloning and sequence analysis of the gene encoding LipL41, a surface-exposed lipoprotein of pathogenic *Leptospira* species. Infect Immun 1996;64:2322-30.
20. Blanco DR, Walker EM, Haake DA, Champion CI, Miller JN, Lovett MA. Complement activation limits the rate of in vitro treponemicidal activity and correlates with antibody-mediated aggregation of *Treponema pallidum* rare outer membrane protein. J Immunol 1990;144:1914-21.
21. Jones JD, Bourell KW, Norgard MV, Radolf JD. Membrane topology of *Borrelia burgdorferi* and *Treponema pallidum* lipoproteins. Infect Immun 1995;63:2424-34.
22. Miller JN. Immunity in experimental syphilis. VI. Successful vaccination of rabbits with *Treponema pallidum*, Nichols strain, attenuated by gamma-irradiation. J Immunol 1973;110:1206-15.
23. Blanco DR, Reimann K, Skare JT, Champion CI, Foley D, Exner MM, et al. Isolation of the outer membranes from *Treponema pallidum* and *Treponema vincentii*. J Bacteriol 1994;176:6088-99.
24. Belisle JT, Akins DR, Radolf JD, Norgard MV. Fatty acids of *Borrelia burgdorferi* and *Treponema pallidum* lipoproteins. J Bacteriol 1994;176:2151-7.
25. Radolf JD. *Treponema pallidum* and the quest for outer membrane proteins. Mol Microbiol 1995;16:1067-73.
26. Akins DR, Purcell BK, Mitra MM, Norgard MV, Radolf JD. Lipid modification of the 17-kilodalton membrane immunogen of *Treponema pallidum* determines macrophage activation as well as amphilicity. Infect Immun 1993;61:1201-10.
27. Schouls LM, Mout R, Dekker J, van Embden JDA. Characterization of lipid-modified immunogenic proteins of *Treponema pallidum* expressed in *Escherichia coli*. Microb Pathog 1989;7:175-88.
28. Radolf JD, Robinson EJ, Bourell KW, Akins DR, Porcella SF, Weigel LM, et al. Characterization of outer membranes isolated from *Treponema pallidum*, the syphilis spirochete. Infect Immun 1995;63:4244-52.
29. Hancock REW. Model membrane studies of porin function. In: Inouye M, editor. Bacterial outer membranes as model systems. New York: John Wiley and Sons, 1986.
30. Weiss MS, Abele U, Weckesser J, Welte W, Schiltz E, Schulz GE. Molecular architecture and electrostatic properties of a bacterial porin. Science 1991;254:1627-30.
31. Bishop ND, Leu EJ. Characterization of the porin of *Rhodobacter capsulatus* 3764 in planar lipid bilayers. FEBS Lett 1994;349:69-74.
32. Bellinger-Kawahara C, Horwitz MA. Complement component C3 fixes selectively to the major outer membrane protein (MOMP) of *Legionella pneumophila* and mediates phagocytosis of liposome-MOMP complexes by human monocytes. J Exp Med 1990;172:1201-10.
33. Su H, Watkins NG, Zhang YX, Caldwell HD. *Chlamydia trachomatis*-host cell interactions: role of the chlamydial major outer membrane protein as an adhesin. Infect Immun 1990;58:1017-25.
34. Christodoulides M, Heckels JE. Immunization with a multiple antigen peptide containing defined B- and T-cell epitopes: production of bactericidal antibodies against group B *Neisseria meningitidis*. Microbiol 1994;140:2951-60.
35. Elkins C, Sparling PF. Outer membrane proteins of *Neisseria gonorrhoeae.* In: Ayoub EM, Cassell GH, Branche WC, Henry TJ, editors. Microbial determinants of virulence and host response. Washington (DC): American Society for Microbiology, 1990.
36. Murphy TF, Bartos LC. Human bactericidal antibody response to outer membrane protein P2 of nontypeable *Haemophilus influenzae.* Infect Immun 1988;56:2673-9.
37. Saukkonen K, Abdillahi H, Poolman JT, Leinonen M. Protective efficacy of monoclonal antibodies to class 1 and class 3 outer membrane proteins of *Neisseria meningitidis* B:15:P1.16 in infant rat infection model: new prospects for vaccine development. Microb Pathog 1987;3:261-7.
38. Blanco DR, Champion CI, Exner MM, Erdjument-Bromage H, Hancock REW, Tempst P, et al. Porin activity and sequence analysis of a 31-kilodalton *Treponema pallidum* subsp. *pallidum* rare outer membrane protein (Tromp1). J Bacteriol 1995;177:3556-62.
39. Carbonetti NH, Simnad VI, Seifert HS, So M, Sparling PF. Genetics of protein I of *Neisseria gonorrhoeae*: construction of hybrid porins. Proc Natl Acad Sci USA 1988;85:6841-5.
40. Woodruff WA, Parr TR Jr, Hancock REW, Hanne LF, Nicas TI, Iglewski BH. Expression in *Escherichia coli* and function of *Pseudomonas aeruginosa* outer membrane porin protein F. J Bacteriol 1986;167:473-9.
41. Bishop NH, Miller JN. Humoral immunity in experimental syphilis: II. The relationship of neutralizing factors in immune serum to acquired resistance. J Immunol 1976;117:197-207.

42. Alder JD, Friess L, Tengowski M, Schell RF. Phagocytosis of opsonized *Treponema pallidum* subsp. *pallidum* proceeds slowly. Infect Immun 1990;58:1167-73.
43. Lukehart SA, Miller JN. Demonstration of the in vitro phagocytosis of *Treponema pallidum* by rabbit peritoneal macrophages. J Immunol 1978;121:2014-24.
44. Blanco DR, Champion CI, Exner MM, Shang ES, Skare JT, Hancock REW, et al. Recombinant *Treponema pallidum* rare outer membrane protein 1 (Tromp1) expressed in *Escherichia coli* has porin activity and surface antigenic exposure. J Bacteriol 1996;178:6685-92.
45. Rocque WJ, Coughlin RT, McGroarty EJ. Lipopolysaccharide tightly bound to porin monomers and trimers from *Escherichia coli* K-12. J Bacteriol 1987;169:4003-10.
46. Champion CI, Blanco DR, Erdjument-Bromage H, Exner MM, Hancock REW, Tempst P, et al. Sequence analysis and recombinant expression of a 28-kDa *Treponema pallidum* subsp. *pallidum* rare outer membrane protein (Tromp2). J Bacteriol. In press.
47. Bishop NH, Miller JN. Humoral immune mechanisms in acquired syphilis. In: Schell RF, Musher DM, editors. Pathogenesis and immunology of treponemal infection. New York: Marcel Dekker, 1983:241-69.
